# Real-world Safety and Effectiveness of Insulin Glargine 300 U/mL in Participants with Type 2 Diabetes Mellitus During the Period of Ramadan in Four Countries (Egypt, Jordan, Lebanon, and Turkey): A Prospective Observational Study

**DOI:** 10.2174/1573399820666230811152520

**Published:** 2024-02-14

**Authors:** Mohamed Hassanein, Inass Shaltout, Rachid Malek, Samir Assaad Khalil, Hajar Ballout, Firas Annabi, Mark Shereen

**Affiliations:** 1 Dubai Hospital, Dubai Health Authority, Dubai, United Arab Emirates;; 2 Internal Medicine Department, Cairo University, Cairo, Egypt;; 3 Internal Medicine Department, CHU Mohamed Saadna Abdennour, Se´tif, Algeria;; 4 Department of Internal Medicine, Unit of Diabetes, Lipidology & Metabolism, Alexandria Faculty of Medicine, Alexandria, Egypt;; 5 Private Clinic, Al Rassoul Al Aazam Hospital, Beirut, Lebanon;; 6 Private Clinic, Islamic Hospital, Amman, Jordan;; 7 Department of Medical Affairs, Sanofi, Cairo, Egypt

**Keywords:** Diabetes, type-2 diabetes mellitus, insulin, glargine, ramadan, randomized clinical trials

## Abstract

**Aim:**

This non-interventional observational study aimed to describe the clinical outcomes of patients with T2DM treated with Gla-300 during the period of Ramadan.

**Background:**

Type 2 diabetes mellitus (T2DM) patients who decide to fast during the holy month of Ramadan face several challenges in achieving glycemic control without increasing the risk of hypoglycemia. Insulin glargine-300 (Gla-300) has well-established safety and efficacy in improving glycemic control in multiple randomized clinical trials (RCTs). However, limited evidence is available regarding its safety and effectiveness during fasting.

**Objective:**

The objective of this study was to assess the safety and clinical outcomes of insulin glargine-300 (Gla-300) in T2DM patients before, during, and after Ramadan.

**Methods:**

We conducted a prospective, observational, non-comparative, multicenter study on patients with T2DM currently treated with Gla-300 who planned to fast and continue on Gla-300 during Ramadan in four countries (Egypt, Jordan, Lebanon, and Turkey). The study outcomes included the change in glycemic parameters and incidence of hypoglycemia before, during, and after Ramadan.

**Results:**

One hundred and forty T2DM patients were included. Nearly 61% of the included patients had a duration of diabetes of <10 years. The mean Gla-300 daily doses during the pre-Ramadan, Ramadan, and post-Ramadan periods were 22.2 ±7.4, 20.4 ±7.5, and 22.5 ±4.7 IU, respectively. The mean change values from pre-Ramadan to Ramadan and post-Ramadan were -1.7 ±6.9 IU and 0.5 ±4.7 IU, respectively, among the included patients. The mean HbA1c decreased during the study period initiating from 7.9% ±1.4% pre-Ramadan to 6.9% ±0.4% post-Ramadan. The overall HBA1c target value was 6.9% ±0.4%, while the HbA1c target was achieved by 29 patients (21.9%). The mean fasting blood glucose (FPG) showed a reduction from baseline value in the post-Ramadan period by -0.9 ±2.3 mmol/L. Five patients (3.57%) had symptomatic documented hypoglycemia during Ramadan, and none was considered to have severe hypoglycemia.

**Conclusion:**

Our study showed that insulin Gla-300 maintained the glycemic control of T2DM patients who decided to fast during the holy month of Ramadan without increasing the risk of hypoglycemia. Regular self-monitoring of blood glucose levels during Ramadan is highly recommended to avoid possible complications.

## 1 INTRODUCTION

It is estimated that there are more than 2 billion Muslims worldwide, representing about 25% of the world population [1]. Fasting in the holy month of Ramadan every year is an obligation for all healthy Muslims around the world. Muslims observe absolute fasting (no food or drinks are allowed) from dusk till dawn throughout the entire month. Certain people with medical conditions that can make fasting harmful to their health are exempted from fasting [2]. However, many Muslims prefer to continue fasting against medical advice and religious permission given to them [3].

Because of the long periods of fasting, which may reach up to 18 hours in some countries, patients with type 2 diabetes mellitus (T2DM) who insist on fasting during Ramadan expose themselves to a higher risk of acute complications, including hypoglycemia, hyperglycemia, and dehydration, which may be complicated by diabetic ketoacidosis [4]. Diabetes control is a tough challenge for both the patients and their treating physicians during stressful times and long periods of fasting. As per the 2019 International Diabetes Federation Atlas 9^th^ Edition report, out of 463 million Muslims with diabetes, almost 112 million people choose to fast the entire month of Ramadan [5]. Previous studies have investigated the effect of fasting on body systems and the biochemical changes occurring during Ramadan for both euglycemic and diabetic patients [6-12]. Most of these studies have reported a little change in diabetic control and a reduction in both the lipid profile and body weight of diabetic patients [11, 12]. Good dietary control alone has been shown to improve and stabilize the fasting blood sugar level [12, 13]. Oral antidiabetic drugs (OADs), such as repaglinide, glibenclamide, and gliclazide modified-release, are used effectively for T2DM patients during fasting [12, 14-16]. Also, insulin and its derivatives may be used for both types of diabetes (T1DM or T2DM) [17].

Insulin is one of the treatment options that has been used in combination or alone in patients with T2DM. Long-acting forms of insulin are preferred in the treatment over the short-acting forms as they project a lower frequency of injections, higher patient compliance, and higher diabetic control. Insulin glargine U300 (Gla-300) is one of the long-acting insulin forms that has proven its efficacy in lowering HbA1c levels, providing an effective replacement for basal insulin and protection from the risk of hypoglycemia [18, 19].

The objectives of diabetes management during Ramadan are no different from regular days, and the main goals include symptomatic control, maintaining normal glucose levels, and preventing acute complications. This non-interventional observational study aimed to describe the clinical outcomes in patients with T2DM treated with Gla-300 during the period of Ramadan.

## 2 PATIENTS AND METHODS

### 2.1 Study Design, Setting, and Eligibility Criteria

This prospective, observational, non-comparative, multicenter study was conducted on T2DM patients treated with Gla-300 in Egypt, Jordan, Lebanon, and Turkey prior to, during, and after Ramadan. These four countries were selected because this region has the same fasting hours and similar demographics and culture. The Ramadan was between 5^th^ May and 4^th^ June, 2019. This study is a part of a large-scale multinational study conducted at 64 centers across 11 countries (Kuwait, Qatar, Saudi Arabia, United Arab Emirates, Jordan, Lebanon, Turkey, Egypt, India, Pakistan, and Canada) to describe the clinical outcomes in patients with T2DM, treated with Gla-300 before, during, and after the period of Ramadan [20]. Adult patients (≥ 18 years old) with T2DM were included if they planned to fast for at least 15 days during Ramadan and continue the Gla-300 therapy throughout Ramadan. We excluded patients on basal-bolus/premix insulin in the six months before starting Gla-300, patients with any experimental medication usage within one month before the selection visit, and patients with either hypersensitivity or intolerance to Gla-300 treatment or any of the excipients. Pregnant and lactating women were also excluded. The study protocol was approved by the Research and Ethics Committee of the included centers. A written informed consent was obtained from each participant before study enrollment after explaining the study methodology and objectives. Sites using or planning to use Gla-300 for patients with T2DM during Ramadan and routinely assessing HbA1c at least every six months were invited to participate in the study.

### 2.2 Data Collection

Baseline characteristics data were collected from each included patient, including demographic data, diabetes medical history, and history of diseases other than diabetes. Patients’ demographic data included gender, age, body mass index (BMI), and social profile. The details of diabetic history were collected, including duration of diabetes, concomitant use of antidiabetic drugs, and diabetes-associated complications and comorbidities. Data on medication taken at the study entry and during the study were recorded. Regarding Gla-300, we collected the following data: Start date, current dose, time of injection, and insulin adjustment algorithm recommended by the physician in the three time-point periods. The total period of observation for each patient ranged between 3 and 5 months. Glucose levels, basal insulin dosages, occurrences of symptomatic hypoglycemia, and days with or without fasting, were all recorded. We recorded the hypoglycemia and adverse events (AEs) that occurred one month after Ramadan if the patient visit was delayed more than one month after Ramadan. Patients discontinuing the Gla-300 treatment were observed until the end of the study. The physicians evaluated the International Diabetes Federation-Diabetes and Ramadan (IDF-DAR) categories for “very high”, “high”, and “moderate/low-risk” in diabetic patients who fasted throughout Ramadan and applied them to estimate the risk level of the included individuals [21].

The risk associated with fasting was evaluated according to the guidelines available at the time of the study and according to the interpretation of the investigator.

Insulin glargine 300 U/mL in Solostar Pen (Toujeo®) was prescribed as the routine practice in real-world settings; Dose adjustments during Ramadan were made as per the treating physician and guided by the IDF-DAR practical guidelines as well as patient characteristics [21]. The study participants were assessed at the following time points at study sites: Pre-Ramadan period (1-3 months prior to Ramadan) to match real-life practice as any change in HbA1c is still indicative of glycemic control, Ramadan period (1 month), and post-Ramadan period (1 month) (Supplementary Fig. **S1**).

### 2.3 Study Endpoints

The primary objective of the current study was to report the percentage of T2DM patients experiencing at least one episode of severe and/or symptomatic documented hypoglycemia with self-monitored plasma glucose (SMPG) equal to or less than 70 mg/dL in the three-time points, with the Ramadan period serving as the primary endpoint. The secondary objectives were (1) the percentage of patients experiencing at least one episode of severe and/or symptomatic documented hypoglycemia with SMPG less than 54 mg/dL, and (2) the incidence rate of severe and/or symptomatic documented hypoglycemia with SMPG equal or less than 70 mg/dL and less than 54 mg/dL over the three timepoints and across different times a day. Throughout the pre-Ramadan and post-Ramadan periods, assessments were conducted in the daytime (from 06:00 am to 11:59 pm) and nocturnal (from 12:00 am to 05:59 am). While in the Ramadan period, we assessed the fasting hours (*i.e*., between the meal before dawn and the meal at sunset) as well as the non-fasting hours. Mean changes in HbA1c, fasting plasma glucose (FPG), SMPG, mean change in body weight at the post-Ramadan period compared to the pre-Ramadan period, mean fasting SMPG just prior to iftar throughout the Ramadan period, and changes in Gla-300 dosage from pre-Ramadan period to other time points, were assessed as secondary efficacy endpoints. We documented the AEs, such as hyperglycemic episodes and serious AEs.

### 2.4 Sample Size Calculation

The sample size was based on the primary objective of describing the number of patients experiencing at least 1 episode of severe and/or symptomatic documented hypoglycemia with PG≤70 mg/dL (3.9 mmol/L) during the Ramadan period with sufficient precision (calculation based on the 2-sided 95% confidence interval [CI] for the percentage of patients).

To reach an absolute precision of 4% (20% relative precision), assuming that the percentage of patients with at least one episode of severe and/or symptomatic documented hypoglycemia with PG ≤70 mg/dL (3.9 mmol/L) will be approximately 20% during the Ramadan period, 385 patients would be needed. For pre-/post-Ramadan periods, where the proportion of patients with at least 1 episode of severe and/or symptomatic documented hypoglycemia with PG ≤70 mg/dL (3.9 mmol/L) is expected to be lower (between 5% and 10%), the total number of patients used to describe the hypoglycemia occurrence during Ramadan (N=385) would give an absolute precision from 2.2% to 3%.

Given the study duration and expected loss to follow-up (approximately 10% of patients), the recommendation for this study was to include 430 patients.

### 2.5 Statistical Analysis

Data were gathered during the recruitment visit, as well as throughout the three time points. Continuous data have been presented as mean ± standard deviation (±SD), while the qualitative data have been presented as frequencies (n) and percentages (%). All analyses were performed using Statistical Package of Social Science (SPSS) version 22.

## 3 RESULTS

### 3.1 Patients’ Demographics and Baseline Characteristics

A total of 140 T2DM patients (63 female and 77 male) were included in our study. The mean age was 54.1 ±10.3 and the majority of the patients were aged <65 years (n=122, 87.14%). Overall, 57.1% of the patients had a university/higher educational degree and 59.3% were full-time employees. The mean BMI was 29.9 ±5.5 Kg/m^2^; the majority of the included patients (n= 61, 43.57%) had a BMI≥30. DM-related complications or comorbidities were reported in one-quarter of the included patients (n= 36, 25.71%). Peripheral diabetic neuropathy was reported in 26 patients (18.57%), while diabetic retinopathy was reported in 13 patients (9.29%). Four patients (2.86%) had impaired renal function, three patients (2.14%) had peripheral vascular disease and a history of recurrent hypoglycemia, and at least one macrovascular complication was reported in 5 patients (3.57%). The level of risk associated with fasting, according to the investigator, was low/moderate in 107 patients (76.43%), high in 30 patients (21.43%), and very high in one patient (0.71%). Furthermore, among all the included patients (493), causes for drop-out were patient decision (10; 2.0%), loss to follow-up (14; 2.8%), and other reasons (3; 0.6%) (Table **1**).

### 3.2 Disease Characteristics

The mean age of patients at the onset of diabetes was 45.3±9.9 years and the duration of diabetes was 8.9±5.9 years. Nearly 61% of the included patients had a duration of diabetes of <10 years. Of the 63 females, only 7 patients (5%) had a history of gestational diabetes. 98 patients (70%) received ≥two previous non-insulin antidiabetic treatments within one month before Ramadan. The mean current dose of Gla-300 (U) was 21.4 ±7.6IU. The recommended way of adjusting Gla-300 was physician-driven among 113 patients (80.71%). The recommended dose increment for one adjustment step was two units among 120 patients (85.71%), while it was four units among the remaining patients (n= 18, 12.86%). The disease characteristics and recommended frequency of dose adjustment are shown in Table **2**.

### 3.3 Adverse events

During the follow-up, hypoglycemic events showed a rare incidence during the study period, as shown in Supplementary Tables **S1-S3**. Supplementary Table **S1** shows the number of symptomatic hypoglycemia events (any time of the day, nocturnal, daytime) per patient-month of follow-up per type of hypoglycemia. Only one patient had symptomatic hypoglycemia during the pre-Ramadan period. The number of patients with at least one severe and/or symptomatic documented hypoglycemia event with plasma glucose (PG) ≤ 3.9 mmol/L (70 mg/dL) and < 3.0 mmol/L (54 mg/dL) by period is reported in Supplementary Table **S2**. Five patients, with a percentage of 3.57%, had symptomatic documented hypoglycemia with PG≤ 3.9 mmol/L (70 mg/dL) during the Ramadan period, and none of them were considered as

having severe hypoglycemia. Supplementary Table **S3** describes the patients with at least one symptomatic hypoglycemia event (any time of the day, between Suhur and Iftar, between Iftar and Suhur) per type of hypoglycemia event during the Ramadan period. None of the included patients had AEs leading to Gla-300 discontinuation or AEs leading to death.

### 3.4 Gla-300 Daily Dose

As shown in Table **3**, the mean Gla-300 daily doses during the pre-Ramadan, Ramadan, and post-Ramadan periods were 22.2 ±7.4, 20.4 ±7.5, and 22.5 ±4.7IU, respectively. The mean change values from pre-Ramadan to Ramadan and post-Ramadan were -1.7 ±6.9 IU and 0.5 ±4.7 IU, respectively, among the included patients.

### 3.5 Treatment Effectiveness Outcomes

The mean HbA1c decreased during the study period initiating from 7.9% ±1.4% pre-Ramadan to 6.9% ±0.4% post-Ramadan. The overall HbA1c target value was 6.9% ±0.4%, while the HbA1c target was achieved by 29 patients (21.9%). The mean FPG and fasting SMPG showed a reduction from baseline values in the post-Ramadan period. The mean FPG change and fasting SMPG change were -0.9 ±2.3mmol/L and -0.4 ±1.5mmol/L, respectively (Table **4**).

## 4 DISCUSSION

Fasting during the holy month of Ramadan is one of the five pillars of Islam; however, fasting, especially intermittent fasting, could be performed for non-religious health reasons, such as losing weight or changing dietary habits. A management plan including dose adjustment and regular monitoring during Ramadan is substantial for all diabetic patients before fasting in order to avoid the risk of hypoglycemic or hyperglycemic events during Ramadan. Treatment-related problems, such as medication non-adherence, fear of diabetes complications, and possible treatment-related AEs, should be addressed and reviewed before fasting.

The ORION study aimed to assess the real-world safety and clinical outcomes of Gla-300 among T2DM patients willing to fast during Ramadan. We included 140 T2DM patients from four countries in the Middle East. Most of the included participants fasted the entire month of Ramadan despite the increased risk of hypoglycemia. Treatment with Gla-300 showed stable glycemic control during Ramadan and post-Ramadan. Also, FPG and fasting SMPG measurements showed a drop in their post-Ramadan values. A minimal Gla-300 dose adjustment was reported during Ramadan and post-Ramadan periods. The Gla-300 showed a high safety profile, as hypoglycemic events showed a rare incidence during the study period. Only five patients, with a percentage of 3.57%, had symptomatic documented hypoglycemia with PG≤ 3.9 mmol/L (70 mg/dL) during Ramadan, and none were considered as having severe hypoglycemia. No documented severe hypoglycemic events with SMPG < 70 or < 54 mg were reported during the pre-Ramadan and post-Ramadan periods. The non-extended period of fasting in Egypt contributes significantly to this lower risk of hypoglycemia. Fasting hours are not consistent across different countries. For example, fasting hours in 2019 were nearly 15 hours in Egypt, compared to 18.75 hours in Canada, 16.5 hours in Algeria, 14.6 hours in Saudi Arabia, and 17.3 hours in Germany. The low incidence rate of hypoglycemia supports the safety of Gla-300 during Ramadan, especially since 117 patients (83.57%) were on two or more non-insulin antidiabetic treatments before Ramadan. Our results align with two small observational studies that found Gla-300 use to have no significant effect on the incidence of hypoglycemia compared to non-fasting diabetic patients or patients on OADs [22, 23].

The IDF-DAR guidelines state that T2DM patients at “very high-risk” or “high risk” of developing hypoglycemia should not fast during Ramadan [21]. However, these practical guidelines are not fully practiced as almost 20% of our patients were at high risk, yet they completed their fasting. Patients were recommended to use insulin injections during iftar and lower their insulin dose during Ramadan as per DAR clinical practice guidelines [21].

Our study supports the current body of evidence for managing T2DM in patients willing to fast during Ramadan. Two large epidemiological studies, CREED and DAR-MENA, have investigated the characteristics and current management plan of T2DM during Ramadan in North Africa and the Middle East region [24, 25]. A lower number of patients were using insulin during fasting in both CREED and DAR-MENA studies compared to ORION; however, both studies showed a higher incidence of hypoglycemic events [24, 25]. The incidence rate of hypoglycemia during Ramadan was 8.8% in the CREED study [24] and 10.4% in the DAR-MENA study [25]. Also, our study results regarding clinical outcomes and safety profiles matched data of the previously published clinical trials, including EDITION 3 and BRIGHT trials [26, 27]. The HbA1c% in EDITION 3 and BRIGHT trials exhibited a significant improvement than our study, yet it may be related to the small number of our included patients, the short follow-up period, and the burden of fasting on the clinical outcomes. Insulin safety and efficacy during Ramadan depend on many factors, including the fasting period, patients’ education, lifestyle modifications (diet and regular exercise), dose adjustment, and insulin timing [28].

### 4.1 Strengths and Limitations

The ORION study is the first prospective non-interventional real-world study to collect data related to safety and clinical outcomes of basal Gla-300 use in T2DM patients during Ramadan in four countries (Egypt, Jordan, Lebanon, and Turkey). Our study reflects that patients on Gla-300 who fasted during the holy month of Ramadan did not experience significant impairment in their glycemic control or increased risk of hypoglycemia. The relatively long period of the study (>3 months) enabled us to detect HbA1c fluctuations during pre-Ramadan, Ramadan, and post-Ramadan periods. Also, it enabled us to detect any AEs related to drug use. Most of our included patients completed the whole follow-up period without any treatment discontinuation. On the other hand, major limitations of our study include the lack of randomization due to the descriptive nature of the study and the relatively small number of the included patients (140 patients). Also, the follow-up period was inconsistent for all patients (ranging from 3 to 5 months). Moreover, the absence of a comparator arm in our study may create some selection bias and limit our results. Additionally, a major confounder could exist due to different dietary patterns during the non-fasting and fasting months in different countries. The narrow scope of our included patients, T2DM patients receiving Gla-300 and willing to continue on it during Ramadan, and the absence of any very high-risk patients according to IDF-DAR guidelines have limited the ability to generalize our results to patients with other types of diabetes and reflect the safety of Gla-300 among the very high-risk group.

## CONCLUSION

Our study showed that insulin Gla-300 maintained the glycemic control of T2DM patients who decided to fast during the holy month of Ramadan without increasing the risk of hypoglycemia. Regular self-monitoring of blood glucose levels during Ramadan is highly recommended to avoid possible complications.

## Figures and Tables

**Table 1:** Patients’ demographics and baseline characteristics of the included patients.

-	-	Egypt (n= 36)	Jordan (n= 40)	Lebanon (n= 30)	Turkey (n= 34)	All (n= 140)
Age (years)	Mean (SD)	53.4 (7.3)	52.2 (12.8)	54.6 (7.9)	56.7 (11.4)	54.1 (10.3)
Age group (years) [n (%)]	<65	34 (94.4%)	35 (87.5%)	28 (93.3%)	25 (73.5%)	122 (87.14%)
	[65-75]	2 (5.6%)	3 (7.5%)	2 (6.7%)	9 (26.5%)	16 (11.43%)
	≥75	0 (0%)	2 (5.0%)	0 (0.0%)	0 (0.0%)	2 (1.43%)
Gender [n (%)]	Female	15 (41.7%)	16 (40.0%)	13 (43.3%)	19 (55.9%)	63 (45%)
	Male	21 (58.3%)	24 (60.0%)	17 (56.7%)	15 (44.1%)	77 (55%)
Bodyweight (kg)	Mean (SD)	90.8 (10.1)	81.9 (14.9)	76.9 (13.3)	85.3 (13.0)	83.9 (13.8)
BMI (kg/m^2^) [n (%)]	<25	2 (5.6%)	9 (22.5%)	9 (30.0%)	5 (14.7%)	25 (17.86%)
	[25-30]	12 (33.3%)	19 (47.5%)	17 (56.7%)	6 (17.6%)	54 (38.57%)
	[30-40]	21 (58.3%)	11 (27.5%)	4 (13.3%)	19 (55.9%)	55 (39.29%)
	≥40	1 (2.8%)	1 (2.5%)	0 (0.0%)	4 (11.8%)	6 (4.29%)
Educational level [n (%)]	Primary education not completed	0 (0%)	1 (2.5%)	0 (0.0%)	7 (20.6%)	8 (5.71%)
	Primary	0 (0%)	3 (7.5%)	4 (13.3%)	14 (41.2%)	21 (15%)
	Secondary	1 (2.8%)	5 (12.5%)	16 (53.3%)	6 (17.6%)	28 (20%)
	University/Higher educational	35 (97.2%)	29 (72.5%)	10 (33.3%)	6 (17.6%)	80 (57.14%)
	Unknown	0 (0%)	2 (5.0%)	0 (0.0%)	0 (0.0%)	2 (1.43%)
	Doesn’t want to answer	0 (0%)	0 (0.0%)	0 (0.0%)	1 (2.9%)	1 (0.71%)
Employment status [n (%)]	Full time	28 (77.8%)	27 (67.5%)	19 (63.3%)	9 (26.5%)	83 (59.29%)
	Part time	0 (0%)	0 (0.0%)	0 (0.0%)	1 (2.9%)	1 (0.71%)
	Not employed	6 (16.7%)	7 (17.5%)	10 (33.3%)	14 (41.2%)	37 (26.43%)
	Student	0 (0%)	0 (0.0%)	0 (0.0%)	0 (0.0%)	0 (0%)
	Retired	2 (5.6%)	5 (12.5%)	1 (3.3%)	4 (11.8%)	12 (8.57%)
	Incapacity	0 (0%)	0 (0.0%)	0 (0.0%)	5 (14.7%)	5 (3.57%)
	Unknown	0 (0%)	1 (2.5%)	0 (0.0%)	1 (2.9%)	2 (1.43%)
Work disability due to diabetes [n (%)]*¶*	Yes	0 (0%)	0 (0.0%)	0 (0.0%)	0 (0.0%)	0 (0%)
	No	6 (100.0%)	7 (100.0%)	10 (100.0%)	20 (100.0%)	43 (30.71%)
Life style [n (%)]	Alone	1 (2.8%)	0 (0.0%)	1 (3.3%)	0 (0.0%)	2 (1.43%)
	With another adult	32 (88.9%)	40 (100.0%)	29 (96.7%)	33 (97.1%)	134 (95.71%)
	In an institution or community	3 (8.3%)	0 (0.0%)	0 (0.0%)	1 (2.9%)	4 (2.86%)
Prior and concomitant medication	Any insulin anti-diabetic medication	0 (0%)	0 (0.0%)	0 (0.0%)	0 (0.0%)	0 (0%)
	Any antidiabetic non-insulin medication	31 (86.1%)	40 (100.0%)	18 (60.0%)	28 (82.4%)	117 (83.57%)
Diabetes complication and comorbidities history	Any complication or comorbidity	12 (33.3%)	9 (22.5%)	4 (13.3%)	11 (32.4%)	36 (25.71%)
	Diabetic Neuropathy [n (%)]	11 (30.6%)	7 (17.5%)	2 (6.7%)	6 (17.6%)	26 (18.57%)
	*If Yes [n (%)]:*	*-*	-	-	-	-
	Autonomic 0 (0%)	-	0 (0%)	0 (0.0%)	0 (0.0%)	0 (0.0%)
	Peripheral	11 (100.0%)	7 (100.0%)	2 (100.0%)	6 (100.0%)	26 (18.57%)
	Diabetic Retinopathy [n (%)]	7 (19.4%)	3 (7.5%)	0 (0.0%)	3 (8.8%)	13 (9.29%)
	*If yes, leading to blindness [n (%)]:*			*-*	-	-
	Yes 0 (0.0%) 0 (0%) 0 (0%)		-	-	0 (0%)	0 (0.0%)
	No	7 (100.0%)	3 (100.0%)	0 (0.0%)	3 (100.0%)	13 (9.29%)
	Impaired Renal Function [n (%)] *¶¶*	0 (0%)	1 (100.0%)	1 (3.3%)	2 (5.9%)	4 (2.86%)
	Peripheral Vascular Disease [n (%)]	1 (2.8%)	2 (5.0%)	0 (0.0%)	0 (0.0%)	3 (2.14%)
	History Of Hypoglycemia Unawareness [n (%)]	0 (0%)	0 (0.0%)	0 (0.0%)	0 (0.0%)	0 (0%)
	History Of Recurrent Hypoglycemia [n (%)]	0 (0%)	1 (2.5%)	0 (0.0%)	2 (5.9%)	3 (2.14%)
	At least one macrovascular complication ** [n (%)]	0 (0%)	1 (2.5%)	0 (0.0%)	4 (11.8%)	5 (3.57%)
Level of risk associated with fasting according to the investigator [n (%)]	Moderate/low risk	29 (80.6%)	22 (55.0%)	29 (96.7%)	27 (84.4%)	107 (76.43%)
	High risk	7 (19.4%)	17 (42.5%)	1 (3.3%)	5 (15.6%)	30 (21.43%)
	Very high risk	0 (0%)	1 (2.5%)	0 (0.0%)	0 (0.0%)	1 (0.71%)

**Note:** ¶ If “Part-time” or “Not employed” or “Incapacity” answered in employment status; ** Macrovascular complication includes the following events: coronary heart disease; Myocardial Infarction; Heart Failure; Stroke; Transient ischemic attack; ¶¶ All events were related to diabetes

**Table 2:** Disease characteristics and dose adjustment among the included patients.

**-**	**-**	**Egypt (n= 36)**	**Jordan (n= 40)**	**Lebanon (n= 30)**	**Turkey (n= 34)**	**All (n= 140)**
**Duration of diabetes (years)**	Mean (SD)	9.9 (5.9)	9.5 (6.4)	6.3 (2.9)	9.2 (5.2)	8.9 (5.5)
**Duration of diabetes (years) [n (%)]**	<10	17 (47.2%)	22 (55.0%)	26 (86.7%)	21 (61.8%)	86 (61.43%)
	≥10	19 (52.8%)	18 (45.0%)	4 (13.3%)	13 (38.2%)	54 (38.57%)
**Age at onset of diabetes (years)**	Mean (SD)	43.4 (6.9)	42.6 (11.6)	48.1 (6.7)	47.7 (11.8)	45.3 (9.9)
**History of gestational diabetes [n (%)] #**	Yes	1 (6.7%)	4 (25.0%)	0 (0.0%)	2 (10.5%)	7 (5%)
	No	14 (93.3%)	12 (75.0%)	13 (100.0%)	17 (89.5%)	56 (40%)
**Time elapsed since any insulin received for the first time (years)**	Mean (SD)	0.5 (0.2)	1.0 (2.2)	2.2 (2.2)	3.1 (2.9)	2.5 (3.8)
**Duration of insulin glargine U300 treatment (months)**	Mean (SD)	5.2 (2.1)	3.9 (2.0)	7.1 (4.6)	5.8 (4.5)	5.4 (3.6)
**Number of previous noninsulin antidiabetic treatments [n (%)] ####**	0	5 (13.9%)	0 (0.0%)	12 (40.0%)	6 (17.6%)	23 (16.43%)
	1	5 (13.9%)	5 (12.5%)	5 (16.7%)	4 (11.8%)	19 (13.57%)
	2	14 (38.9%)	16 (40.0%)	5 (16.7%)	16 (47.1%)	51 (36.43%)
	>2	12 (33.3%)	19 (47.5%)	8 (26.7%)	8 (23.5%)	47 (33.57%)
**Previous noninsulin antidiabetic treatments [n (%)] ####**	Sulfonylureas	26 (72.2%)	20 (50.0%)	13 (43.3%)	1 (2.9%)	60 (42.86%)
	Biguanides	22 (61.1%)	35 (87.5%)	13 (43.3%)	25 (73.5%)	95 (67.86%)
	DDP-4 inhibitors	8 (22.2%)	33 (82.5%)	12 (40.0%)	20 (58.8%)	73 (52.14%)
	Thiazolidinediones	8 (22.2%)	3 (7.5%)	1 (3.3%)	1 (2.9%)	13 (9.29%)
	SGLT-2 inhibitors	4 (11.1%)	10 (25.0%)	1 (3.3%)	8 (23.5%)	23 (16.43%)
	GLP-1 RA	1 (2.8%)	0 (0.0%)	0 (0.0%)	4 (11.8%)	5 (3.57%)
	Other	0 (0%)	0 (0.0%)	0 (0.0%)	0 (0.0%)	0 (0%)
	Alpha-glucosidase inhibitors	0 (0%)	0 (0.0%)	0 (0.0%)	1 (2.9%)	1 (0.71%)
	Glinides	0 (0%)	0 (0.0%)	0 (0.0%)	1 (2.9%)	1 (0.71%)
**Number of previous noninsulin antidiabetic treatments within one month prior to Ramadan [n (%)]**	0	5 (13.9%)	0 (0.0%)	12 (40.0%)	6 (17.6%)	23 (16.43%)
	1	5 (13.9%)	5 (12.5%)	5 (16.7%)	4 (11.8%)	19 (13.57%)
	2	14 (38.9%)	16 (40.0%)	5 (16.7%)	16 (47.1%)	51 (36.43%)
	>2	12 (33.3%)	19 (47.5%)	8 (26.7%)	8 (23.5%)	47 (33.57%)
**SU or glinides intake within one month prior to Ramadan [n (%)]**	Yes	26 (72.2%)	20 (50.0%)	13 (43.3%)	2 (5.9%)	61 (43.57%)
	No	10 (27.8%)	20 (50.0%)	17 (56.7%)	32 (94.1%)	79 (56.43%)
**Current dose of insulin glargine U300 (U)**	Mean (SD)	19.7 (5.6)	23.7 (7.1)	20.7 (8.5)	21.0 (8.8)	21.4 (7.6)
**The recommended way of adjustment of insulin glargine U300 [n (%)]**	Patient-driven ###	11 (30.6%)	9 (23.1%)	0 (0.0%)	6 (17.6%)	26 (18.57%)
	Physician-driven ##	25 (69.4%)	30 (76.9%)	30 (100.0%)	28 (82.4%)	113 (80.71%)
**Recommended frequency of adjustment [n (%)]**	More than 1 week (1 to 6 days)	6 (16.7%)	28 (71.8%)	16 (53.3%)	8 (23.5%)	58 (41.43%)
	Every week	6 (16.7%)	5 (12.8%)	13 (43.3%)	3 (8.8%)	27 (19.29%)
	Less than every week and more than every 2 weeks	0 (0%)	0 (0.0%)	0 (0.0%)	2 (5.9%)	2 (1.43%)
	Less than every 2 weeks and more than every month	12 (33.3%)	3 (7.7%)	1 (3.3%)	5 (14.7%)	21 (15%)
	Less than every month	12 (33.3%)	3 (7.7%)	0 (0.0%)	16 (47.1%)	31 (22.14%)
**Recommended dose increment for one adjustment step [n (%)]**	< 2 Units	0 (0%)	1 (2.6%)	0 (0.0%)	0 (0.0%)	1 (0.71%)
	2 Units	27 (75.0%)	31 (79.5%)	30 (100.0%)	32 (94.1%)	120 (85.71%)
	3 Units	0 (0%)	0 (0.0%)	0 (0.0%)	0 (0.0%)	0 (0%)
	4 Units	9 (25.0%)	7 (17.9%)	0 (0.0%)	2 (5.9%)	18 (12.86%)
	6 Units	0 (0%)	0 (0.0%)	0 (0.0%)	0 (0.0%)	0 (0%)
	> 6 Units	0 (0%)	0 (0.0%)	0 (0.0%)	0 (0.0%)	0 (0%)
**Lowest value ** **recommended**	> 70 mg/dL (3.9 mmol/L)	0 (0%)	6 (15.0%)	3 (10.0%)	15 (44.1%)	24 (17.14%)
	> 80 mg/dL (4.4 mmol/L)	24 (66.7%)	21 (52.5%)	15 (50.0%)	4 (11.8%)	64 (45.71%)
	> 90 mg/dL (5.0 mmol/L)	12 (33.3%)	13 (32.5%)	12 (40.0%)	15 (44.1%)	52 (37.14%)
**Highest value ** **recommended**	< 100 mg/dL (5.6 mmol/L)	0 (0%)	16 (40.0%)	12 (40.0%)	0 (0.0%)	28 (20%)
	< 110 mg/dL (6.1 mmol/L)	7 (19.4%)	4 (10.0%)	4 (13.3%)	10 (29.4%)	25 (17.86%)
	< 120 mg/dL (6.7 mmol/L)	19 (52.8%)	2 (5.0%)	8 (26.7%)	5 (14.7%)	34 (24.29%)
	< 130 mg/dL (7.2 mmol/L)	8 (22.2%)	14 (35.0%)	6 (20.0%)	2 (5.9%)	30 (21.43%)
	< 140 mg/dL (7.8 mmol/L)	2 (5.6%)	4 (10.0%)	0 (0.0%)	17 (50.0%)	23 (16.43%)
**Diabetic ketoacidosis within 3 months prior to Ramadan**	Yes	0 (0%)	0 (0.0%)	0 (0.0%)	0 (0.0%)	0 (0%)

**Note:** # Percentage calculated over the number of females in the eligible population; ## The adjustment is adapted by the physician at each contact -visit or phone- with the patient; ### patient has been instructed by the physician to self-adjust their basal insulin; #### Prior medications are limited to those taken within the 8 weeks prior to selection visit

**Table 3:** Insulin glargine U300 (Gla-300) daily dose change during the study.

-	**Egypt (n= 36)**	**Jordan (n= 40)**	**Lebanon (n= 30)**	**Turkey (n= 34)**	**Total (n =140)**
**Pre-Ramadan period**	21.5 (5.4)	24.4 (7.0)	20.8 (8.5)	21.6 (8.6)	22.2 (7.4)
**Ramadan period**	21.8 (5.6)	24.0 (7.8)	15.6 (5.8)	19.3 (7.9)	20.4 (7.5)
**Change from pre-Ramadan to Ramadan**	0.22 (1.33)	-0.28 (8.81)	-5.23 (5.12)	-2.17 (8.11)	-1.7 (6.9)
**Relative change from pre-Ramadan to Ramadan**	0.010 (0.061)	0.028 (0.328)	-0.217 (0.179)	-0.059 (0.250)	-0.06 (0.3)
**Relative change from pre-Ramadan to Ramadan (%)**					
**>0**	1 (2.8%)	9 (25.0%)	0 (0.0%)	11 (36.7%)	21 (15%)
**[0, -15]**	35 (97.2%)	20 (55.6%)	11 (36.7%)	10 (33.3%)	76 (54.3%)
**[-15, -30]**	0 (0%)	5 (13.9%)	6 (20.0%)	5 (16.7%)	16 (11.4%)
**<-30**	0 (0%)	2 (5.6%)	13 (43.3%)	4 (13.3%)	19 (13.6%)
**Post-Ramadan**	21.8 (5.7)	26.3 (8.0)	20.1 (8.2)	21.3 (9.0)	22.5 (8)
**Change from pre-Ramadan to post-Ramadan**	0.25 (1.71)	2.06 (7.62)	-0.67 (2.25)	-0.13 (3.51)	0.5 (4.7)
**Relative change from pre-Ramadan to post-Ramadan**	0.012 (0.073)	0.118 (0.319)	-0.023 (0.107)	0.004 (0.154)	0.033 (0.2)

**Table 4:** Baseline and endpoint data of HbA1c, FPG, and fasting SMPG among the studied patients.

-	Egypt (n= 36)		Jordan (n= 40)		Lebanon (n= 30)		Turkey (n= 34)		Total (n =140)	
	**HbA1c (mmol/mol)**	**HbA1c (%)**	**HbA1c (mmol/mol)**	**HbA1c (%)**	**HbA1c (mmol/mol)**	**HbA1c (%)**	**HbA1c (mmol/mol)**	**HbA1c (%)**	**HbA1c (mmol/mol)**	**HbA1c (%)**
**Pre-Ramadan period**	60.5 (12.5)	7.68 (1.14)	64.5 (16.5)	8.06 (1.51)	58.7 (15.2)	7.52 (1.39)	68.7 (14.9)	8.44 (1.36)	62.9 (15.2)	7.9 (1.4)
**HBA1C target value**	52.3 (2.6)	6.94 (0.24)	50.1 (5.4)	6.74 (0.49)	50.8 (4.5)	6.80 (0.41)	53.4 (5.2)	7.03 (0.48)	51.6 (4.7)	6.9 (0.4)
**Patients at HbA1c target [n (%)]**										
**Yes**	11 (30.6%)	11 (30.6%)	0 (0.0%)	0 (0.0%)	14 (46.7%)	14 (46.7%)	4 (14.3%)	4 (14.3%)	29 (21.9%)	29 (21.9%)
**No**	25 (69.4%)	25 (69.4%)	38 (100.0%)	38 (100.0%)	16 (53.3%)	16 (53.3%)	24 (85.7%)	24 (85.7%)	103 (78.1%)	103 (78.1%)
**Post-Ramadan period**	54.6 (7.8)	7.15 (0.71)	58.3 (8.9)	7.49 (0.81)	58.1 (12.8)	7.47 (1.17)	58.6 (14.7)	7.51 (1.35)	57.3 (11.1)	7.4 (1)
**Change from pre-Ramadan period**	-5.9 (11.3)	-0.54 (1.03)	-5.9 (10.7)	-0.54 (0.98)	-0.5 (3.6)	-0.05 (0.33)	-10.4 (15.9)	-0.95 (1.45)	-5.5 (11.4)	-0.51 (1.04)
**-**	**FPG (mmol/L)**	**FPG (mg/dL)**	**FPG (mmol/L)**	**FPG (mg/dL)**	**FPG (mmol/L)**	**FPG (mg/dL)**	**FPG (mmol/L)**	**FPG (mg/dL)**	**FPG (mmol/L)**	**FPG (mg/dL)**
**Pre-Ramadan period**	8.06 (2.40)	145.2 (43.3)	8.49 (3.20)	153.0 (57.6)	8.07 (3.52)	145.4 (63.4)	8.99 (2.88)	162.0 (51.9)	8.4 (3)	151.3 (54.1)
**Post-Ramadan period**	6.28 (0.90)	113.2 (16.1)	7.23 (1.06)	130.3 (19.2)	7.47 (2.77)	134.6 (49.8)	8.81 (2.66)	158.8 (48.0)	7.4 (2.1)	132.9 (38.4)
**Change from pre-Ramadan period**	-1.78 (2.15)	-32.0 (38.7)	-0.84 (2.05)	-15.1 (37.0)	-0.60 (1.77)	-10.7 (31.9)	-0.08 (3.07)	-1.5 (55.3)	-0.9 (2.3)	-15.8 (42.1)
**-**	**Fasting SMPG (mmol/L)**	**Fasting SMPG (mg/dL)**	**Fasting SMPG (mmol/L)**	**Fasting SMPG (mg/dL)**	**Fasting SMPG (mmol/L)**	**Fasting SMPG (mg/dL)**	**Fasting SMPG (mmol/L)**	**Fasting SMPG (mg/dL)**	**Fasting SMPG (mmol/L)**	**Fasting SMPG (mg/dL)**
**Pre-Ramadan period, mean (SD)**	7.30 (1.78)	131.5 (32.1)	7.84 (1.99)	141.2 (35.9)	7.74 (2.94)	139.5 (53.0)	7.57 (1.82)	136.3 (32.8)	7.6 (2.1)	137.2 (38.6)
**Post-Ramadan period, mean (SD)**	6.37 (0.79)	114.7 (14.3)	7.25 (1.16)	130.7 (20.8)	7.33 (2.74)	132.0 (49.4)	8.07 (2.56)	145.4 (46.0)	7.2 (2)	129.9 (36)
**Change from pre-Ramadan period, mean (SD)**	-0.93 (1.52)	-16.8 (27.5)	-0.41 (1.64)	-7.5 (29.5)	-0.41 (1.27)	-7.4 (22.8)	0.18 (1.46)	3.3 (26.3)	-0.4 (1.5)	-7.6 (27.4)
**Fasting SMPG in the evening just before Iftar, mean (SD)**	6.48 (1.06)	116.8 (19.1)	6.08 (0.84)	109.5 (15.1)	8.79 (2.85)	158.4 (51.3)	6.75 (1.56)	121.7 (28.1)	6.8 (1.8)	122.1 (31.8)
**Fasting SMPG whatever the time, mean (SD)**	6.48 (1.06)	116.8 (19.1)	6.08 (0.84)	109.5 (15.1)	8.11 (2.37)	146.2 (42.6)	7.03 (1.87)	126.7 (33.7)	6.9 (1.8)	123.7 (31.6)

## Data Availability

The data and supportive information are available within the article.

## LIST OF ABBREVIATIONS

AEs: Adverse Events
BMI: Body Mass Index
CI: Confidence Interval
FPG: Fasting Blood Glucose
Gla-300: Insulin Glargine-300
IDF-DAR: International Diabetes Federation-Diabetes and Ramadan
OADs: Oral Antidiabetic Drugs
PG: Plasma Glucose
RCTs: Randomized Clinical Trials
SMPG: Self-Monitored Plasma Glucose
SPSS: Statistical Package Of Social Science
T1DM: Type 1 Diabetes Mellitus
T2DM: Type 2 Diabetes Mellitus
