# Supplementary material for: Real-world Safety and Effectiveness of Insulin Glargine 300 U/mL in Participants with Type 2 Diabetes Mellitus During the Period of Ramadan in Four Countries (Egypt, Jordan, Lebanon, and Turkey): A Prospective Observational Study
Source: Curr Diabetes Rev. 2024 Feb 14;20(5):E110823219694. doi: 10.2174/1573399820666230811152520 (PMC11071651; doi:10.2174/1573399820666230811152520)
Supplement: Supplementary file 1 [file CDR-20-E110823219694_SD1.pdf]

Supplementary Material

Real-world Safety and Effectiveness of Insulin Glargine 300 U/mL in Participants with Type 2 Diabetes Mellitus During the Period of Ramadan in Four Countries (Egypt, Jordan, Lebanon, and Turkey): A Prospective Observational Study

Mohamed Hassanein[1,*], Inass Shaltout[2], Rachid Malek[3], Samir Assaad Khalil[4], Hajar Ballout[5], Firas Annabi[6] and Mark Shereen[7]

[1]Dubai Hospital, Dubai Health Authority, Dubai, United Arab Emirates; [2]Internal Medicine Department, Cairo University, Cairo, Egypt; [3]Internal Medicine Department, CHU Mohamed Saadna Abdennour, Se´tif, Algeria; [4]Department of Internal Medicine, Unit of Diabetes, Lipidology & Metabolism, Alexandria Faculty of Medicine, Alexandria, Egypt; [5]Private Clinic - Al Rassoul Al Aazam Hospital, Beirut, Lebanon; [6]Private Clinic - Islamic Hospital, Amman, Jordan; [7]Department of Medical Affairs, Sanofi, Cairo, Egypt

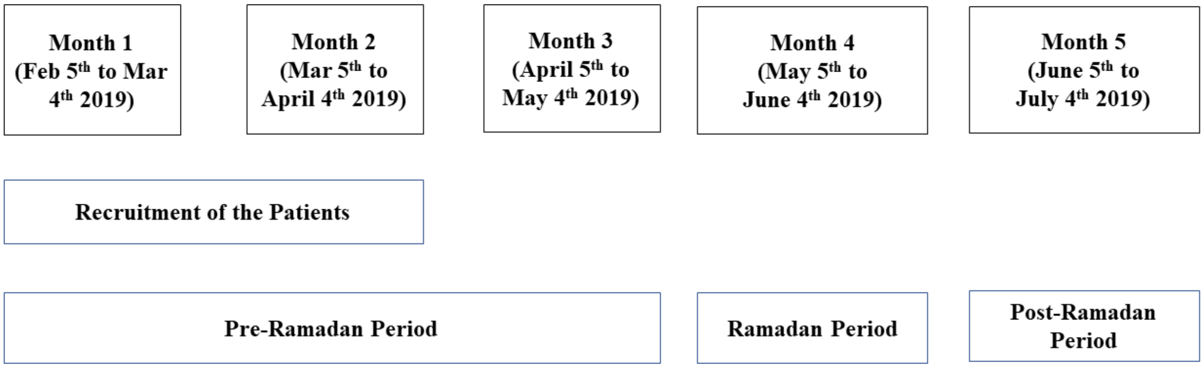

Fig. (S1). Study design.

Table S1. Number of symptomatic hypoglycemia events (any time of the day, nocturnal, daytime) per patient-month of follow-up per type of hypoglycemia.

		Egypt (n= 36)				Jordan (n= 40)				Lebanon (n= 30)				Turkey (n= 34)				Total (n= 140)			
		All	Nocturnal (clock time)	Nocturnal (sleep status)	Day time	All	Nocturnal (clock time)	Nocturnal (sleep status)	Day time	All	Nocturnal (clock time)	Nocturnal (sleep status)	Day time	All	Nocturnal (clock time)	Nocturnal (sleep status)	Day time	All	Nocturnal (clock time)	Nocturnal (sleep status)	Day time
Any symptomatic hypoglycemia event	Pre-Ramadan	0 (0.0)	0 (0.0)	0 (0.0)	0 (0.0)	0 (0.0)	0 (0.0)	0 (0.0)	0 (0.0)	1 (0.017)	0 (0.0)	1 (0.017)	1 (0.017)	0 (0.0)	0 (0.0)	0 (0.0)	0 (0.0)	1 (0.7%)	0 (0%)	1 (0.7%)	1 (0.7%)
	Post-Ramadan	0 (0.0)	0 (0.0)	0 (0.0)	0 (0.0)	0 (0.0)	0 (0.0)	0 (0.0)	0 (0.0)	0 (0.0)	0 (0.0)	0 (0.0)	0 (0.0)	0 (0.0)	0 (0.0)	0 (0.0)	0 (0.0)	0 (0%)	0 (0%)	0 (0%)	0 (0%)
Severe hypoglycemia and/or documented symptomatic hypoglycemia																					
≤ 3.9 mmol/L (70 mg/dL)	Pre-Ramadan	0 (0.0)	0 (0.0)	0 (0.0)	0 (0.0)	0 (0.0)	0 (0.0)	0 (0.0)	0 (0.0)	1 (0.017)	0 (0.0)	1 (0.017)	1 (0.017)	0 (0.0)	0 (0.0)	0 (0.0)	0 (0.0)	1 (0.7%)	0 (0%)	1 (0.7%)	1 (0.7%)
	Post-Ramadan	0 (0.0)	0 (0.0)	0 (0.0)	0 (0.0)	0 (0.0)	0 (0.0)	0 (0.0)	0 (0.0)	0 (0.0)	0 (0.0)	0 (0.0)	0 (0.0)	0 (0.0)	0 (0.0)	0 (0.0)	0 (0.0)	0 (0%)	0 (0%)	0 (0%)	0 (0%)

**< 3.0 mmol/L (54 mg/dL)**	Pre-Rama-dan	0 (0.0)	0 (0.0)	0 (0.0)	0 (0.0)	0 (0.0)	0 (0.0)	0 (0.0)	0 (0.0)	0 (0.0)	0 (0.0)	0 (0.0)	0 (0.0)	0 (0.0)	0 (0.0)	0 (0.0)	0 (0.0)	0 (0%)	0 (0%)	0 (0%)	0 (0%)
	Post-Rama-dan	0 (0.0)	0 (0.0)	0 (0.0)	0 (0.0)	0 (0.0)	0 (0.0)	0 (0.0)	0 (0.0)	0 (0.0)	0 (0.0)	0 (0.0)	0 (0.0)	0 (0.0)	0 (0.0)	0 (0.0)	0 (0.0)	0 (0%)	0 (0%)	0 (0%)	0 (0%)
**Severe hypoglyce-mia**	Pre-Rama-dan	0 (0.0)	0 (0.0)	0 (0.0)	0 (0.0)	0 (0.0)	0 (0.0)	0 (0.0)	0 (0.0)	0 (0.0)	0 (0.0)	0 (0.0)	0 (0.0)	0 (0.0)	0 (0.0)	0 (0.0)	0 (0.0)	0 (0%)	0 (0%)	0 (0%)	0 (0%)
	Post-Rama-dan	0 (0.0)	0 (0.0)	0 (0.0)	0 (0.0)	0 (0.0)	0 (0.0)	0 (0.0)	0 (0.0)	0 (0.0)	0 (0.0)	0 (0.0)	0 (0.0)	0 (0.0)	0 (0.0)	0 (0.0)	0 (0.0)	0 (0%)	0 (0%)	0 (0%)	0 (0%)
**Documented symptomatic hypoglycemia**																					
**≤ 3.9 mmol/L (70 mg/dL)**	Pre-Rama-dan	0 (0.0)	0 (0.0)	0 (0.0)	0 (0.0)	0 (0.0)	0 (0.0)	0 (0.0)	0 (0.0)	1 (0.017)	0 (0.0)	1 (0.017)	1 (0.017)	0 (0.0)	0 (0.0)	0 (0.0)	0 (0.0)	1 (0.7%)	0 (0%)	1 (0.7%)	1 (0.7%)
	Post-Rama-dan	0 (0.0)	0 (0.0)	0 (0.0)	0 (0.0)	0 (0.0)	0 (0.0)	0 (0.0)	0 (0.0)	0 (0.0)	0 (0.0)	0 (0.0)	0 (0.0)	0 (0.0)	0 (0.0)	0 (0.0)	0 (0.0)	0 (0%)	0 (0%)	0 (0%)	0 (0%)
**< 3.0 mmol/L (54 mg/dL)**	Pre-Rama-dan	0 (0.0)	0 (0.0)	0 (0.0)	0 (0.0)	0 (0.0)	0 (0.0)	0 (0.0)	0 (0.0)	0 (0.0)	0 (0.0)	0 (0.0)	0 (0.0)	0 (0.0)	0 (0.0)	0 (0.0)	0 (0.0)	0 (0%)	0 (0%)	0 (0%)	0 (0%)
	Post-Rama-dan	0 (0.0)	0 (0.0)	0 (0.0)	0 (0.0)	0 (0.0)	0 (0.0)	0 (0.0)	0 (0.0)	0 (0.0)	0 (0.0)	0 (0.0)	0 (0.0)	0 (0.0)	0 (0.0)	0 (0.0)	0 (0.0)	0 (0%)	0 (0%)	0 (0%)	0 (0%)
**Probable symptomatic hypoglyce-mia**	Pre-Rama-dan	0 (0.0)	0 (0.0)	0 (0.0)	0 (0.0)	0 (0.0)	0 (0.0)	0 (0.0)	0 (0.0)	0 (0.0)	0 (0.0)	0 (0.0)	0 (0.0)	0 (0.0)	0 (0.0)	0 (0.0)	0 (0.0)	0 (0%)	0 (0%)	0 (0%)	0 (0%)
	Post-Rama-dan	0 (0.0)	0 (0.0)	0 (0.0)	0 (0.0)	0 (0.0)	0 (0.0)	0 (0.0)	0 (0.0)	0 (0.0)	0 (0.0)	0 (0.0)	0 (0.0)	0 (0.0)	0 (0.0)	0 (0.0)	0 (0.0)	0 (0%)	0 (0%)	0 (0%)	0 (0%)
**Relative hypoglyce-mia**	Pre-Rama-dan	0 (0.0)	0 (0.0)	0 (0.0)	0 (0.0)	0 (0.0)	0 (0.0)	0 (0.0)	0 (0.0)	0 (0.0)	0 (0.0)	0 (0.0)	0 (0.0)	0 (0.0)	0 (0.0)	0 (0.0)	0 (0.0)	0 (0%)	0 (0%)	0 (0%)	0 (0%)
	Post-Rama-dan	0 (0.0)	0 (0.0)	0 (0.0)	0 (0.0)	0 (0.0)	0 (0.0)	0 (0.0)	0 (0.0)	0 (0.0)	0 (0.0)	0 (0.0)	0 (0.0)	0 (0.0)	0 (0.0)	0 (0.0)	0 (0.0)	0 (0%)	0 (0%)	0 (0%)	0 (0%)
**Not classified – Severity unknown**	Pre-Rama-dan	0 (0.0)	0 (0.0)	0 (0.0)	0 (0.0)	0 (0.0)	0 (0.0)	0 (0.0)	0 (0.0)	0 (0.0)	0 (0.0)	0 (0.0)	0 (0.0)	0 (0.0)	0 (0.0)	0 (0.0)	0 (0.0)	0 (0%)	0 (0%)	0 (0%)	0 (0%)
	Post-Rama-dan	0 (0.0)	0 (0.0)	0 (0.0)	0 (0.0)	0 (0.0)	0 (0.0)	0 (0.0)	0 (0.0)	0 (0.0)	0 (0.0)	0 (0.0)	0 (0.0)	0 (0.0)	0 (0.0)	0 (0.0)	0 (0.0)	0 (0%)	0 (0%)	0 (0%)	0 (0%)

Probable symptomatic hypoglycemia is an event during which symptoms of hypoglycemia are not accompanied by a plasma glucose determination, but was presumably caused by a plasma glucose concentration less than or equal to 3.9 mmol/L (≤70 mg/dL); symptoms treated with oral carbohydrate without a test of plasma glucose; Relative hypoglycemia is an event during which the person with diabetes reports any of the typical symptoms of hypoglycemia, and interprets the symptoms as indicative of hypoglycemia, but with a measured plasma glucose concentration greater than 3.9 mmol/L (>70 mg/dL); Not classified – Severity unknown are those in which classification item countermeasure administration is missing, and countermeasure is answered as Yes (i.e., no idea if the patient was not capable of Treating Self and required assistance) and the severity of the hypoglycemia could not be determined; n(rate)= number of symptomatic hypoglycemias and rate of events per patient-month

Table S2. Number (%) of patients with at least one severe and/or symptomatic documented hypoglycemia event with plasma glucose (PG) ≤ 3.9 mmol/L (70 mg/dL) and < 3.0 mmol/L (54 mg/dL) by period.

	Egypt (N=36)		Jordan (N=40)		Lebanon (N=30)		Turkey (N=34)		Total (n= 140)	
**Severe and/or symptomatic documented hypoglycemia with plasma glucose (PG)**	**≤ 3.9 mmol/L (70 mg/dL)**	**< 3.0 mmol/L (54 mg/dL)**	**≤ 3.9 mmol/L (70 mg/dL)**	**< 3.0 mmol/L (54 mg/dL)**	**≤ 3.9 mmol/L (70 mg/dL)**	**< 3.0 mmol/L (54 mg/dL)**	**≤ 3.9 mmol/L (70 mg/dL)**	**< 3.0 mmol/L (54 mg/dL)**	**≤ 3.9 mmol/L (70 mg/dL)**	**< 3.0 mmol/L (54 mg/dL)**
Pre-Ramadan whole period	0 (0.0%)	0 (0.0%)	0 (0.0%)	0 (0.0%)	1 (3.3%)	0 (0.0%)	0 (0.0%)	0 (0.0%)	1 (0.7%)	0 (0%)
Pre-Ramadan last month period	0 (0.0%)	0 (0.0%)	0 (0.0%)	0 (0.0%)	0 (0.0%)	0 (0.0%)	0 (0.0%)	0 (0.0%)	0 (0%)	0 (0%)

Ramadan period	1 (2.8%)	0 (0.0%)	4 (10.0%)	0 (0.0%)	0 (0.0%)	0 (0.0%)	0 (0.0%)	0 (0.0%)	5 (3.6%)	0 (0%)
Post-Ramadan whole period	0 (0.0%)	0 (0.0%)	0 (0.0%)	0 (0.0%)	0 (0.0%)	0 (0.0%)	0 (0.0%)	0 (0.0%)	0 (0%)	0 (0%)
Post-Ramadan first month period	0 (0.0%)	0 (0.0%)	0 (0.0%)	0 (0.0%)	0 (0.0%)	0 (0.0%)	0 (0.0%)	0 (0.0%)	0 (0%)	0 (0%)

Table S3. Number (%) of patients with at least one symptomatic hypoglycemia event (any time of the day, between Suhur and Iftar, between Iftar and Suhur) per type of hypoglycemia event during the Ramadan period.

	Egypt (n= 36)			Jordan (n= 40)			Lebanon (n= 30)			Turkey (n= 34)			Total (n= 140)		
	**All**	**Between Suhur and Iftar**	**Between Iftar and Suhur**	**All**	**Between Suhur and Iftar**	**Between Iftar and Suhur**	**All**	**Between Suhur and Iftar**	**Between Iftar and Suhur**	**All**	**Between Suhur and Iftar**	**Between Iftar and Suhur**	**All**	**Between Suhur and Iftar**	**Between Iftar and Suhur**
**Any symptomatic hypoglycemia event**	1 (2.8%)	1 (2.8%)	0 (0.0%)	4 (10.0%)	4 (10.0%)	0 (0.0%)	0 (0.0%)	0 (0.0%)	0 (0.0%)	0 (0.0%)	0 (0.0%)	0 (0.0%)	5	5	0
**Severe hypoglycemia and/or documented symptomatic hypoglycemia**															
**≤ 3.9 mmol/L (70 mg/dL)**	1 (2.8%)	1 (2.8%)	0 (0.0%)	4 (10.0%)	4 (10.0%)	0 (0.0%)	0 (0.0%)	0 (0.0%)	0 (0.0%)	0 (0.0%)	0 (0.0%)	0 (0.0%)	5 (3.6%)	5 (3.6%)	0 (0%)
**< 3.0 mmol/L (54 mg/dL)**	0 (0.0%)	0 (0.0%)	0 (0.0%)	0 (0.0%)	0 (0.0%)	0 (0.0%)	0 (0.0%)	0 (0.0%)	0 (0.0%)	0 (0.0%)	0 (0.0%)	0 (0.0%)	0 (0%)	0 (0%)	0 (0%)
**Severe hypoglycemia**	0 (0.0%)	0 (0.0%)	0 (0.0%)	0 (0.0%)	0 (0.0%)	0 (0.0%)	0 (0.0%)	0 (0.0%)	0 (0.0%)	0 (0.0%)	0 (0.0%)	0 (0.0%)	0 (0%)	0 (0%)	0 (0%)
**Documented symptomatic hypoglycemia**															
**≤ 3.9 mmol/L (70 mg/dL)**	1 (2.8%)	1 (2.8%)	0 (0.0%)	4 (10.0%)	4 (10.0%)	0 (0.0%)	0 (0.0%)	0 (0.0%)	0 (0.0%)	0 (0.0%)	0 (0.0%)	0 (0.0%)	5 (3.6%)	5 (3.6%)	0 (0%)
**< 3.0 mmol/L (54 mg/dL)**	0 (0.0%)	0 (0.0%)	0 (0.0%)	0 (0.0%)	0 (0.0%)	0 (0.0%)	0 (0.0%)	0 (0.0%)	0 (0.0%)	0 (0.0%)	0 (0.0%)	0 (0.0%)	0 (0%)	0 (0%)	0 (0%)
**Probable symptomatic hypoglycemia**	0 (0.0%)	0 (0.0%)	0 (0.0%)	0 (0.0%)	0 (0.0%)	0 (0.0%)	0 (0.0%)	0 (0.0%)	0 (0.0%)	0 (0.0%)	0 (0.0%)	0 (0.0%)	0 (0%)	0 (0%)	0 (0%)
**Relative hypoglycemia**	0 (0.0%)	0 (0.0%)	0 (0.0%)	0 (0.0%)	0 (0.0%)	0 (0.0%)	0 (0.0%)	0 (0.0%)	0 (0.0%)	0 (0.0%)	0 (0.0%)	0 (0.0%)	0 (0%)	0 (0%)	0 (0%)
**Not classified – Severity unknown**	0 (0.0%)	0 (0.0%)	0 (0.0%)	0 (0.0%)	0 (0.0%)	0 (0.0%)	0 (0.0%)	0 (0.0%)	0 (0.0%)	0 (0.0%)	0 (0.0%)	0 (0.0%)	0 (0%)	0 (0%)	0 (0%)
